# The Emerging Role of Ferroptosis in EBV-Associated Cancer: Implications for Cancer Therapy

**DOI:** 10.3390/biology13070543

**Published:** 2024-07-18

**Authors:** Shan He, Cheng Luo, Feng Shi, Jianhua Zhou, Li Shang

**Affiliations:** 1 Key Laboratory of Carcinogenesis and Cancer Invasion of Chinese Ministry of Education, XiangYa Hospital, Central South University, Changsha 410078, China; 13173817031@163.com (S.H.); chengluo622@163.com (C.L.); shi_feng@csu.edu.cn (F.S.); zhoujh15@163.com (J.Z.); 2 Department of Pathology, National Clinical Research Center for Geriatric Disorders/XiangYa Hospital, Central South University, Changsha 410078, China; 3 Key Laboratory of Carcinogenesis of National Health Commission, Cancer Research Institute and School of Basic Medical Science, Xiangya School of Medicine, Central South University, Changsha 410078, China

**Keywords:** ferroptosis, Epstein–Barr Virus, programmed cell death, infection-related cancer, cancer therapy

## Abstract

**Simple Summary:**

This review will discuss the connection between ferroptosis and Epstein–Barr Virus as well as its associated cancers. It delves into both known and potential ways in which EBV regulates the ferroptosis signaling pathways and focuses on the potential and prospects of targeting the ferroptosis pathway for the treatment of EBV-related tumors. The review may offer new perspectives for future research directions and clinical therapies.

**Abstract:**

Ferroptosis is a novel and iron-dependent form of programmed cell death, which has been implicated in the pathogenesis of various human cancers. EBV is a well-recognized oncogenic virus that controls multiple signaling pathways within the host cell, including ferroptosis signaling. Recent studies show that inducing ferroptosis could be an efficient therapeutic strategy for EBV-associated tumors. This review will firstly describe the mechanism of ferroptosis, then summarize EBV infection and EBV-associated tumors, as well as the crosstalk between EBV infection and the ferroptosis signaling pathway, and finally discuss the role and potential application of ferroptosis-related reagents in EBV-associated tumors.

## 1. Introduction

Programmed cell death (PCD) plays an important role in tissue homeostasis [1]. Ferroptosis is a new form of PCD, which is regulated in an iron-dependent manner [2,3,4]. Ferroptosis is involved in the pathogenesis of various diseases, such as neurodegenerative diseases, tissue injury, inflammation, and cancers, and has become an area of active research [5].

Activating cell death pathways is an important mechanism for the host in defense against pathogenic evasion [6]. Ferroptosis has been observed in HSV-1 infected cells, in addition to apoptosis and necroptosis [7]. On the other hand, resisting cell death is clearly beneficial to the survival of pathogens. For example, Kaposi’s sarcoma-associated herpesvirus (KSHV)-associated primary effusion lymphoma (PEL) cells showed high resistance to ferroptosis, which contributes to the dissemination and further infection of KSHV in host cells [8].

Epstein–Barr Virus (EBV) is a well-recognized oncogenic virus, which is also known as human herpesvirus type 4 [9,10]. The products of EBV genes, such as LMP1, LMP2 (latent membrane proteins 1/2), EBV nuclear antigens, and EBV-encoded microRNAs, have been found to induce host lipid metabolism and disequilibrium of redox homeostasis, which may play a role in ferroptosis [11,12,13]. Ferroptosis impacts both the progression of various EBV-associated cancers and their resistance to therapy. Recent studies show that inducing ferroptosis in EBV-related cancers can effectively suppress tumor growth and is an effective strategy to reduce resistance to radiotherapy and chemotherapy in EBV-associated cancer [14,15,16,17].

In this review, we first discuss the mechanism of ferroptosis. Then, we discuss EBV infection and EBV-associated tumors, as well as the crosstalk between EBV infection and ferroptosis signaling pathways, including known and potential mechanisms. Finally, we discuss the potential therapeutic significance of targeting ferroptosis in EBV-associated tumors.

## 2. Mechanisms of Ferroptosis

Ferroptosis is caused by iron-dependent unrestricted lipid peroxidation and the subsequent rupture of the cytoplasmic membrane [18]. Morphologically, cells undergoing ferroptosis exhibit marked mitochondrial shrinkage, reduced or even absent mitochondrial cristae, and ruptured outer mitochondrial membranes [19,20]. The biochemistry of ferroptosis is characterized by intracellular GSH depletion, reduced glutathione peroxidase 4 (GPX4) activity, and reactive oxygen species (ROS) production [21,22].

### 2.1. Iron Homeostasis

**Figure 1:**
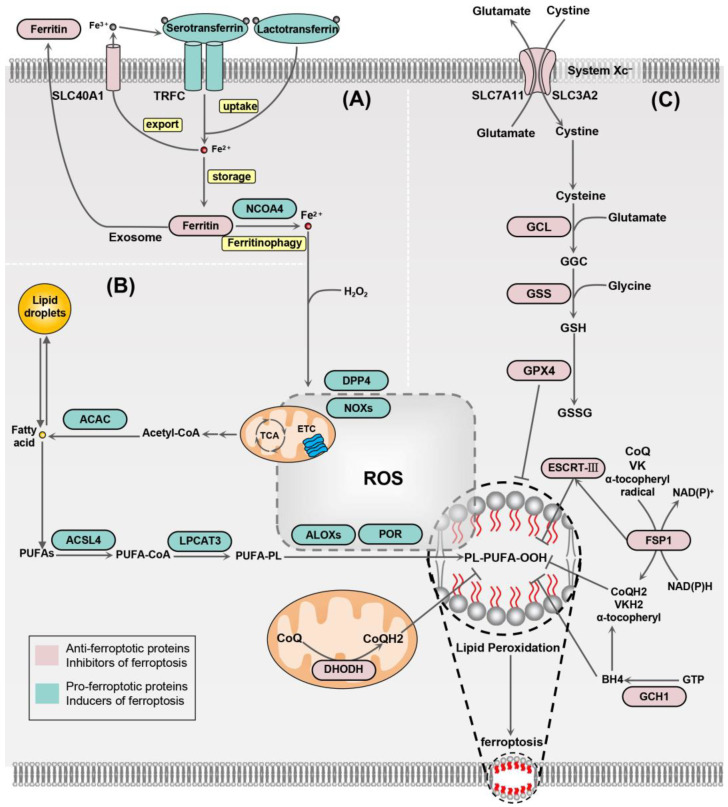
Main mechanisms of ferroptosis. (**A**) Serotransferrin and lactotransferrin are capable of carrying iron ions and transferring them into the cell via the transferrin receptor (TRFC). Ferritin can store intracellular iron ions while working with solute carrier family 40 member 1 (SLC40A1) to transfer iron out of the cell. These proteins, including nuclear receptor coactivator 4 (NCOA4), control ferroptosis by regulating iron homeostasis. (**B**) Acetyl-CoA carboxylase (ACAC)-mediated fatty acid synthesis or fatty acid release from lipid droplets forms polyunsaturated fatty acids (PUFAs), which are incorporated into phospholipids catalyzed by long-chain fatty acid-CoA ligase 4 (ACSL4) and lysophospholipid acyltransferase 3 (LPCAT3), and are further utilized for the formation of lipid hydroperoxides using ROS mediated by lipoxygenases (ALOXs), thereby promoting ferroptosis. (**C**) Cystine/glutamate transporter protein (System Xc^−^) imports cellular cystine while exporting glutamate. Intracellular cystine is reduced to cysteine, which is synthesized into glutathione catalyzed by glutamate–cysteine ligase (GCL) and glutathione synthetase (GSS). GPX4 can utilize glutathione to reduce lipid peroxides to lipid alcohols. The antioxidant system centered on GPX4 is effective in resisting ferroptosis. In addition to this, ferroptosis suppressor protein 1 (FSP1), the endosomal sorting complex required for transport-III (ESCRT-III), and guanosine 5’-triphosphate cyclohydrolase I (GCH1) can also inhibit ferroptosis. DHODH, dihydroorotate dehydrogenase; NOXs, NADPH oxidases; DPP4, dipeptidyl peptidase 4; ETC, electron transport chain; TCA, tricarboxylic acid cycle; VK, vitamin K; VKH2, vitamin K hydroquinone.

Under normal conditions, intracellular iron is in dynamic equilibrium. Iron in the body is primarily absorbed by the small intestine and transported in the blood in the form of Fe^3+^ bound to transferrin. Excess iron is stored by ferritin. The absorption and release of iron in the body are strictly regulated by factors such as the transferrin receptor and hepcidin [23]. Iron within cells is mainly regulated by iron pools in the mitochondria and cytoplasm. When the balance of intracellular iron is disrupted, a condition of iron overload can occur. Iron overload contributes to the execution of ferroptosis mainly by two main mechanisms. First, the Fenton reaction induced by iron may create high levels of ROS. Second, the peroxidation of lipids from free polyunsaturated fatty acids (PUFAs) is catalyzed by the activation of lipoxygenases (LOXs) (Figure 1). Iron uptake is mainly mediated by serotransferrin and lactotransferrin, which translocate iron into the cell via the transferrin receptor (TFRC) [24,25]. Ferritin is the major iron storage protein, which is composed of ferritin heavy (FTH) chains and ferritin light (FTL) chains [26]. Since ferritin is able to rapidly store and release iron steadily over a long time, it is essential to iron homeostasis (Figure 1A). Through the degradation of ferritin, ROS-induced autophagy can enhance ferroptosis [27]. In contrast, exosome-mediated ferritin export is able to inhibit ferroptosis [28].

### 2.2. Lipid Peroxidation

Lipid peroxidation induces ferroptosis by affecting the covalent modification of proteins and nucleic acids and the physical properties of cell membranes [29] (Figure 1B). The primary substrates susceptible to lipid peroxidation are PUFAs [30]. Lipid peroxidation may occur by enzymatic or non-enzymatic catalysis. Enzymatic lipid peroxidation is primarily catalyzed by lipoxygenases (LOXs) and cyclooxygenases (COXs). Non-enzymatic lipid peroxidation is triggered by the direct peroxidation of PUFAs through oxygen radicals [31]. It is worth noting that monounsaturated fatty acids (MUFAs), when substituted for PUFAs in membrane phospholipids, inhibit the accumulation of lipid ROS on the plasma membrane, thereby suppressing ferroptosis [32]. Several membrane electron transport proteins, such as NADPH oxidases (NOXs), contribute to ROS production, which promotes lipid peroxidation as well as ferroptosis. For example, dipeptidyl-peptidase-4 (DPP4) increases ROS production and lipid peroxidation by binding with NOX1, which has been shown to be required for lipid peroxidation in TP53-dependent ferroptosis [33] (Figure 1B).

### 2.3. Antioxidant System

In the process of ferroptosis, oxidative stress plays a crucial role. As mentioned above, iron ions can lead to the production of ROS, particularly the highly reactive hydroxyl radicals generated through the Fenton reaction. These radicals then trigger lipid peroxidation, which induces ferroptosis [34]. The antioxidant system is inherently present in the cell to resist ferroptosis. GPX4 is considered a central inhibitor of ferroptosis that can reduce toxic phospholipid hydroperoxides into non-toxic lipid alcohols [35]. GSH is an important member of the cellular antioxidant system, which is the electron donor of GPX4. FSP1 is capable of reducing phospholipid oxidation in an NAD(P)H-dependent manner and can resist ferroptosis independently of GPX4 [28]. It achieves this by reducing CoQ10 (ubiquinone), vitamin K (VK), and the α-tocopheryl radical, thereby inhibiting ferroptosis [36]. Also, FSP1 can enhance endosomal sorting complexes III (ESCRT-III)-dependent membrane repair, which inhibits ferroptosis [37]. BH4, as an antioxidant in the synthesis of CoQ, prevents the peroxidation of specific phospholipids and thereby inhibits ferroptosis [24]. DHO dehydrogenase (DHODH), another ferroptosis resistance factor, plays a key role in clearing extensive mitochondrial lipid peroxidation by reducing CoQ to CoQH2 independent of GPX4 or FSP1 [25] (Figure 1C).

Oxidative stress can impact multiple intracellular signaling pathways, including the activation of NRF2-dependent antioxidant responses, upregulation of hypoxia-inducible factor 1α (HIF-1α) through PI3K/Akt signaling transduction, and effects on TP53 and AMPK-related signaling pathways [38].

## 3. EBV Infection and EBV-Associated Tumors

EBV, discovered in 1964, is endemic in all human populations. EBV mainly infects B cells and epithelial cells and establishes a bipartite lifecycle after primary infection: latent and lytic phases. Like other herpes viruses, latent infection is the predominant form [39]. When lytic reactivation is induced, a large number of viral genomes are produced and packaged into complete viruses for further transmission [5]. In the latent infection state, EBV expresses only limited gene products in host cells, including EBV nuclear antigen (EBNA), latent membrane proteins (LMPs), and non-coding RNAs (EBERs) [40,41].

EBV is a well-recognized carcinogen that is responsible for 1.5% of all human cancers. EBV infection has been implicated in the etiology of malignant tumors originating from epithelial tissue and lymph. Epithelial cancers mainly include nasopharyngeal carcinoma (NPC), gastric carcinoma (GC), and a very small number of breast and thyroid cancers. EBV-associated lymphomas are predominantly from B cells, including Burkitt’s lymphoma (BL), Hodgkin’s lymphomas (HL), post-transplant lymphoproliferative carcinoma (PTLD), and extranodal NK/T-cell lymphoma (ENKTCL). A few other rare cancers, including smooth muscle sarcoma, primary effusion lymphoma, and lung lymphoepithelioma-like carcinoma (LELC), can also be associated with EBV [42,43,44].

## 4. The Crosstalk between EBV Infection and Ferroptosis Signaling Pathway

**Figure 2:**
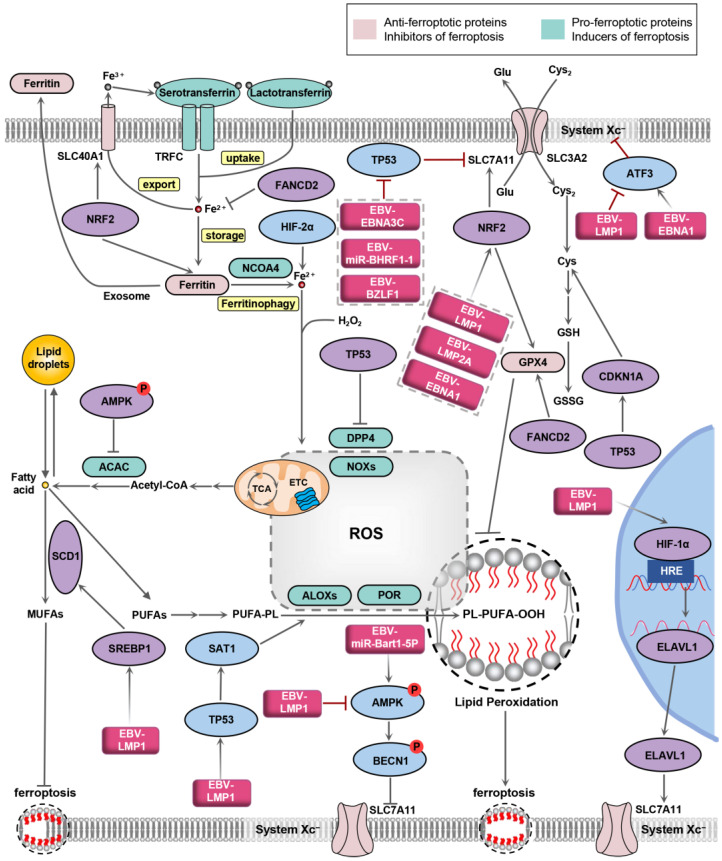
EBV-affected signaling pathways associated with ferroptosis. Oval shapes represent key node proteins of the signaling pathway related to ferroptosis regulated by EBV-encoded products. Squares represent EBV-encoded gene products. NRF2, nuclear factor erythroid 2-related factor-2; HIF-1/2α, hypoxia-inducible factor alpha-subunit protein 1/2; ATF3, activation transcription factor 3; AMPK, AMP-activated protein kinase; SCD1, stearoyl-CoA desaturase; SREBP1, sterol regulatory element-binding protein 1; SAT1, spermidine/spermine N1-acetyltransferase 1; BECN1, beclin 1; ELAVL1, ELAV-like RNA binding protein 1; CDKN1A, cyclin-dependent kinase inhibitor 1A; EBV-LMP1, EBV-encoded latent membrane protein 1; EBV-LMP2A, EBV latent membrane protein 2A; EBNA1, Epstein–Barr nuclear antigen 1; EBNA3C, Epstein–Barr nuclear antigen 3C; FANCD2, Fanconi anemia group D2 protein.

It has been demonstrated that gene products encoded by EBV can regulate multiple signaling pathways in host cells [23]. Some of the signaling pathways hijacked by EBV are involved in ferroptosis. For example, Erastin, a ferroptosis inducer, can increase intracellular iron levels by degrading ferritin in an autophagy-dependent way [27]. EBV infection can decrease ROS-mediated autophagy [45], suggesting that EBV infection may contribute to ferroptosis evasion through decreased ferritinophagy, though additional investigations are needed. Thus, this section focuses on the regulation between EBV infection and ferroptosis signaling (Figure 2).

### 4.1. Nuclear Factor Erythroid 2-Related Factor-2 (NRF2)

NRF2 is a major regulator of the cellular antioxidant response [46]. Firstly, NRF2 is involved in iron metabolism. The iron storage protein, ferritin (which includes FTH and FTL subunits), and the iron transporter protein (SLC40A1) are both controlled by NRF2 at the transcriptional level [47]. In addition to iron metabolism, NRF2 target genes regulate the expression of GSH metabolism-related enzymes, such as GPX4 and SLC7A11 [48,49] (Figure 2). Finally, NRF2 is critical for the regeneration of NADPH. NADPH, as a reducing agent, scavenges ROS and is strongly associated with reduced susceptibility to intracellular ferroptosis [50].

Generating oxidative stress is a major mechanism for host cells to resist virus infection. EBV has been demonstrated to control host cell redox homeostasis. Our previous study showed that EBV-LMP1 is able to increase NRF2 expression in NPC cells [51]. EBV-LMP1 was also found to promote the MEK1/2/NRF2 signaling pathway in lymphoma cells [52]. In B cells with EBV infection, LMP1 and LMP2A activate NRF2 through AKT, thereby promoting the proliferation of host cells [53]. EBV-EBNA1 upregulates SLC7A11 and GPX4 expression and activates NRF2 by degrading Kelch-like ECH-associated protein 1 (Keap1) in a p62-dependent way, which thereby reduces the sensitivity of NPC cells to ferroptosis [17] (Figure 2). Under non-stress conditions, NRF2 is maintained at a low level through Keap1-mediated proteasomal degradation; upon oxidative stress, p62, acting as a receptor and ubiquitin sensor, binds to Keap1 and disrupts the interaction between Keap1 and NRF2, which leads to an elevation in NRF2 expression [54]. In addition, EBV has been found to reduce autophagy, intracellular ROS, and mitochondrial biogenesis, which, in turn, causes the accumulation of p62 and activation of the p62-NRF2 antioxidant response [45,55]. The evidence presented indicates a close association between EBV infection and NRF2. However, it remains unclear whether EBV can truly resist ferroptosis through the NRF2 pathway, and this needs further research in the future.

### 4.2. TP53

TP53 is a well-recognized tumor suppressor gene that plays a pivotal role in many cellular activities, such as cell death [56]. In particular, p53 has both positive and negative effects on ferroptosis (Figure 2). p53 sensitizes cells to ferroptosis by suppressing SLC7A11 expression and cystine uptake [57]. Interestingly, the p53 mutant p53^R175H^ has been found to inhibit ferroptosis by suppressing BACH1-mediated downregulation of SLC7A11 [58]. Spermidine/spermine N1-acetyl-transferase 1 (SAT1) is a transcriptional target of p53, which is known as the rate-limiting enzyme of polyamine catabolism. SAT1 induces lipid peroxidation by enhancing the activity of arachidonate 15-lipoxygenase (ALOX15), while the knockout of SAT1 abrogates p53-induced ferroptosis [59]. Moreover, p53 suppresses ferroptosis by directly reducing DPP4 activity [33]. p53 is also able to prevent ferroptosis in response to cystine deprivation by inducing the expression of CDKN1A/p21 [60]. In addition, the cell cycle arrest caused by p21 can enhance cellular sensitivity to ferroptosis induced by the GPX4 inhibitor [61].

EBV can regulate p53 expression and activity by multiple mechanisms (Figure 2). EBV-EBNA3C is demonstrated to repress the transcriptional regulatory activity of p53 by blocking p53-DNA binding through the N-terminal structural domain [62]. EBV-BZLF1, the immediate-early transcription factor, controls the reactivation of EBV [63]. EBV-BZLF1 can directly interact with p53, which leads to its degradation via the ubiquitin–proteasome pathway [64]. EBV-miR-BHRF1-1 can downregulate p53 protein expression and induce cell proliferation in EBV-associated chronic lymphocytic leukemia [65]. However, the viral protein LMP1 is able to upregulate p53 expression via the H19/miR-675-5p axis [66]. In addition, EBV-LMP1 also contributes to the stability of p53 protein in a ubiquitin-dependent way [67]. Based on the above findings, EBV may have a complex regulatory role in cellular ferroptosis by affecting p53, which is worth exploring.

### 4.3. Activation Transcription Factor 3 (ATF3)

ATF3, a member of the ATF/CREB transcription factor family, is rapidly activated by various cellular stresses, such as oxidative stress [68]. ATF3 has been reported to inhibit the transcription of SLC7A11 and the activity of System Xc^−^, which decreases the intracellular level of GSH and thus promotes erastin-induced ferroptosis [69] (Figure 2). Moreover, ATF3 may block the NRF2/Keap1/xCT signaling pathway, which makes gastric cancer cells sensitive to ferroptosis [70].

In EBV-associated gastric cancer (EBVaGC), EBV-EBNA1 can promote ATF3 expression by binding to the enhancer region upstream of ATF3 [71]. However, LMP1 is demonstrated to inhibit ATF3 protein expression in a dose-dependent manner in NP69, a nasopharyngeal epithelial cell line [72]. Therefore, the effect of EBV on the ATF3 signaling pathway and ATF3-mediated ferroptosis depends on the cellular context.

### 4.4. Sterol Regulatory Element-Binding Protein 1 (SREBP1)

SREBP1 is a central transcription factor of lipid homeostasis, which protects cells from ferroptosis. Activated SREBP1 can promote the transcription of key enzymes in lipogenesis, thereby tightly regulating lipogenesis [73]. Stearoyl-CoA desaturase-1 (SCD1), one of the target genes, is able to convert saturated fatty acids to monounsaturated fatty acids (MUFAs) in an iron-dependent manner [32,74] (Figure 2). Meanwhile, it has been demonstrated that aspirin promotes RSL3-induced ferroptosis by attenuating mTOR/SREBP1/SCD1-mediated lipogenesis [75].

EBV-LMP1 has been shown to increase the expression, maturation, and activation of SREBP1 through the mTOR signaling pathway, which enhances the progression of NPC [73]. Thus, EBV-LMP1 is theoretically able to promote ferroptosis resistance by increasing SREBP1. It seems possible that EBV-LMP1 inhibition could sensitize cells to RSL3-induced ferroptosis, warranting further study.

### 4.5. AMP-Activated Protein Kinase (AMPK)

Rapidly growing cancer cells have an increased demand for energy, and their metabolism is necessarily more rapid, suggesting that cancer cells are more susceptible to energy stress [76]. AMPK is the key sensor of cellular energy status, which is mainly regulated by the AMP/ATP ratio. AMPK controls a large number of key metabolic enzymes through phosphorylation, which protects cells from energy stress by promoting ATP production and reducing ATP consumption [77,78]. Moreover, ROS generated by energy metabolism plays an important role in the process of ferroptosis [79]. In cells with high basal AMPK activation, energy stress-mediated AMPK activation enhances the phosphorylation of ACAC and inhibits the biosynthesis of PUFAs, which contributes to resistance to ferroptosis [80]. AMPK-mediated NRF2 activation can also prevent ferroptosis [81,82]. Autophagy effector protein beclin 1 (BECN1) can regulate various cell processes in an autophagy-dependent or autophagy-independent manner. The ferroptosis inducer erastin activates AMPK through the phosphorylation of AMPKα, which promotes the phosphorylation of BECN1. Phosphorylated BECN1 is able to bind with SLC7A11, which reduces the formation of System Xc^−^ and its activity and thereby accelerates lipid peroxidation in the ferroptosis response [83] (Figure 2).

EBV is known to hijack the energy metabolism of host cells [13]. EBV may be associated with the activation of an adaptive metabolic response to inhibit ferroptosis in host cells. EBV-LMP1 has been demonstrated to reduce the phosphorylation and activity of AMPK [84,85]. However, EBV-miR-Bart1-5P activates the AMPK/mTOR/HIF-1 pathway by targeting the α1 catalytic subunit of AMPK (AMPKα1) in NPC cells [86], suggesting that a feedback mechanism may exist between EBV infection and AMPK. The evidence above suggests that there may be a feedback mechanism between EBV infection and AMPK. EBV may influence cellular energy metabolism through AMPK, which alters the sensitivity of cells to the ferroptosis response.

### 4.6. Hypoxia-Inducible Factors (HIFs)

Hypoxia is involved in tumor formation and treatment resistance, which is a feature of the tumor microenvironment [87]. HIFs are responsible for the primary transcriptional responses to hypoxic stress, including genes involved in hypoxia adaptation and survival. HIFs are heterodimeric complexes consisting of an inducibly expressed α-subunit (such as HIF-1α, HIF-2α, and HIF-3α) and a constitutively expressed β-subunit (HIF-1β). Hypoxic conditions enhance the stabilization of HIFα and the heterodimerization between HIFα and HIF-1β [88]. HIF-1α inhibits the accumulation of ROS and iron by improving the stability of SLC7A11 and, therefore, protects GC cells against ferroptosis [89]. Sorafenib has been reported to trigger ferroptosis of hepatic stellate cells by inhibiting the HIF-1α/SLC7A11 signaling [90] (Figure 2). Interestingly, the activation of HIF-2α improves the sensitivity of colorectal cancer cells to ferroptosis by decreasing GSH production and increasing intracellular ROS and iron levels [91]. The HIF-2α-HILPDA (hypoxia-inducible, lipid droplet-associated protein) axis also promotes ferroptosis by enriching PUFA lipids, the rate-limiting substrates for lipid peroxidation in clear-cell carcinoma cells [92].

EBV infection appears to induce cellular adaptation to hypoxic environments via HIF-related signaling pathways. EBV is able to activate the AKT/HIF-1α axis in NPC and EBVaGC, which promotes vasculogenic mimicry [93]. EBV-LMP1 has been demonstrated to increase the protein expression and transactivation capacity of HIF-1α by enhancing the transcription and stability of HIF-1α mRNA [94]. HIF, in turn, can also regulate EBV infection. HIF-1α activates the transcription of EBV primary latent–lytic switch gene, EBV-BZLF1, by directly binding to its promoter [95]. These studies suggest that EBV infection may affect ferroptosis through the HIF pathway.

## 5. The Role of Ferroptosis in EBV-Associated Tumors

### 5.1. Nasopharyngeal Carcinoma (NPC)

NPC is a malignant carcinoma deriving from the nasopharyngeal epithelium. NPC exhibits a distinct geographical distribution, which is particularly prevalent in East and Southeast Asia and North and East Africa [96]. The World Health Organisation (WHO) classifies NPC into three major pathological subtypes, namely, keratinizing squamous, non-keratinizing, and basaloid squamous, among which the non-keratinizing subtype constitutes most cases in endemic areas (>95%). EBV infection is a major risk factor for NPC, which is detected in 100% of non-keratinizing NPC [40,97]. As EBV in NPC cells is mainly in the latent infection mode, multiple latent gene products, such as LMP1, have been proven to promote carcinogenesis [98].

Recent studies show that ferroptosis and iron metabolism are correlated with NPC carcinogenesis and development. Bioinformatic analyses using TCGA and GEO databases revealed that several ferroptosis-related genes (ATG5, GLS2, and ABCC1) are highly expressed in NPC samples, and this overexpression is associated with poor overall survival [99,100]. 3-Hydroxybutyrate dehydrogenase type 2 (BDH2) is a regulator of intracellular iron homeostasis that shows significantly low expression in NPC. BDH2 inhibits NPC cell proliferation and metastasis by reducing the intracellular iron content [101]. The levels of lipid peroxidation and iron concentration in NPC cells with stem cell-like traits are lower than other NPC cells, while the level of GSH is higher [102].

Therapy resistance in cancer, including chemotherapy and radiotherapy, continues to be a major challenge [103]. Cancer cells can enhance their ability to resist most chemotherapeutic agents by enhancing their redox ability [104]. High levels of oxidative stress may increase the sensitivity to ferroptosis, which provides new therapeutic strategies for therapy-resistant tumors (Table 1) [105]. High expression levels of GPX4 and SLC7A11 caused by EBV increase the resistance of NPC cells to ferroptosis, which contributes to the chemoresistance of EBV-positive NPC cells [17]. Cephalosporin exhibits high specificity and selectivity in inducing NPC cell apoptosis. The inhibition of cancer cell activity by cephalosporin is associated with a significant upregulation of HMOX1, which is known to promote ferroptosis [106]. However, it should be noted that HMOX1 has a dual regulatory effect on ferroptosis. The suppression of ferroptosis can be attributed to the antioxidant activity of HMOX1, while the promotion of ferroptosis may be due to the catalytic degradation of heme to generate increased levels of Fe^2+^ [107]. Cucurbitacin B (CuB) can also promote ferroptosis in NPC cells by reducing GPX4 expression [16]. Lupinol and itracon similarly promote ferroptosis in nasopharyngeal carcinoma cells [102,108]. In addition, the ferroptosis inducer RSL3 and the EGFR monoclonal antibody, Cetuximab, can synergize to impair the cell viability of NPC cells [109]. These data suggest that inducing ferroptosis in NPC cells may enhance chemotherapeutic efficacy.

Radiotherapy is able to eliminate tumor cells through ionizing radiation, which directly leads to DNA damage and indirectly stimulates ROS production [110]. Ionizing radiation generates large amounts of lipid ROS and an excessive accumulation of lipid peroxides, which leads to ferroptosis. Due to their metabolic features, radiation-resistant cells are more susceptible to ferroptosis [111]. Radiotherapy has been shown to activate NRF2, an inhibitor of ferroptosis. The knock-down of NRF2 reduced the resistance of NPC cells to radiotherapy [112]. The Fanconi anemia group D2 protein (FANCD2) is a negative regulator of ferroptosis [113] (Figure 2). Silencing of FANCD2 can also reduce the resistance of NPC cells to ionizing radiation [114]. In addition, several studies have demonstrated that miRNA-regulated ferroptosis serves critical functions in the radioresistance of NPC cells [110]. Taken together, the role of ferroptosis in the resistance to radiotherapy and chemotherapy for nasopharyngeal carcinoma seems to be a potential treatment strategy.

**Table 1:** Compounds that induce or potentially induce ferroptosis in EBV-associated cancers.

Tumor Type	Compounds	Effects	Refs.
NPC	Cucurbitacin B (CuB)	CuB induces widespread lipid peroxidation and downregulates the expression of GPX4, ultimately leading to the ferroptosis of NPC cells.	[16]
NPC	RSL3	RSL3 plays a synergistic role with EGFR monoclonal antibody Cetuximab to inhibit the survival of NPC cells. However, the detailed operation of its mechanism is not fully clear.	[109]
NPC	Lupeol	Lupeol promotes the release of iron and lipid peroxidation in NPC cells, an effect that can be inhibited by the ferroptosis inhibitor Fer-1; at specific dosages, Lupeol suppresses the levels of GSH and GPX4, demonstrating the potential to induce ferroptosis.	[108]
NPC	Itraconazole	Itraconazole triggers ferroptosis and reduces cell viability while partially reversing radioresistance in NPC spherocytes.	[102]
NPC	Isoquercitrin	Isoquercitrin inhibits the proliferation of NPC cells and enhances oxidative stress and ferroptosis within these cells by suppressing the AMPK/NF-κB p65 pathway.	[115]
EBVaGC	Quercetin	Compared to EBV-negative gastric cancer cells, Quercetin has a greater effect on EBV-positive GC and can effectively induce the expression of anticancer factors, such as TP53, in cells.	[116,117]
DLBCL	Dimethyl fumarate (DMF)	DMF exhibits antitumor effects on both subtypes of DLBCL by inducing lipid peroxidation to trigger ferroptosis and is associated with high expression of 5-LOX in the germinal center B-like (GCB) DLBCL subtype.	[118]
DLBCL	APR-246	APR-246 induces p53-dependent ferritinophagy in DLBCL cells and triggers ferroptosis in cells carrying wild-type *TP53* or *TP53* mutants.	[119]
DLBCL	Imidazole ketone erastin (IKE)	IKE induces ferroptosis in DLBCL cells both in vitro and in vivo by inhibiting System Xc^−^, leading to GSH depletion and lipid peroxidation.	[120]
DLBCL	BET inhibitors	BET inhibitors sensitize germinal center B-like (GCB) subtype DLBCL cells to ferroptosis induction, and their combined use with ferroptosis inducers, such as RSL3, enhances their cytotoxic effect on DLBCL cells both in vitro and in vivo.	[121]
DLBCL	Artesunate	Artesunate downregulates the levels of GPX4 and FTH1 in DLBCL cells via STAT3, which promotes ROS accumulation and ferroptosis.	[122]
DLBCL	Iron oxide nanoparticles (IONs)	IONs induce ferroptosis in DLBCL cells by accumulating intracellular iron ions and the onset of lipid peroxidation while inhibiting GPX4 and SLC40A1 expression.	[123]
NKTCL	Kayadiol	Kayadiol decreases GSH in NKTCL cells and induces ferroptosis in NKTCL cells by inhibiting SLC7A11 and GPX4 expression through the upregulation of TP53.	[124]
BL	Buthionine sulfoximine (BSO)	BSO significantly increases the level of lipid ROS in EBV-positive BL cells, and Fer-1 and GSH can inhibit BSO-induced BL cell death, indicating that BSO has the potential to induce ferroptosis in BL cells.	[125]
BL	Artesunate	Artesunate induces ferroptosis by depleting GSH through the upregulation of ATF4-related pathways.	[15]

NPC, nasopharyngeal carcinoma; HMOX1, heme oxygenase 1; GSH, glutathione; GPX4, glutathione Peroxidase 4; EBVaGC, EBV-associated gastric cancer; DLBCL, diffuse large B-cell lymphoma; BET, bromodomain and extra-terminal domain; FSP1, ferroptosis suppressor protein 1; STAT3, signal transducer and activator of transcription 3; NKTCL, natural killer cell and T-cell lymphomas; BL, Burkitt’s lymphoma; ATF4, activation transcription factor 4.

### 5.2. EBV-Associated Gastric Cancer (EBVaGC)

EBVaGC, which possesses specific molecular phenotypes and clinical features, was discovered in 1992 [115,126]. Approximately 9% of gastric cancer cases are found to be EBV-associated, which tend to occur in the proximal part of the stomach and are usually adenocarcinoma [98]. Similar to NPC, EBV latent infection is mainly observed in EBVaGC, although LMP1 is not detected in some cases [127].

The EBV-encoded non-coding small RNAs EBER1 and EBER2 are generally highly expressed in EBVaGC cells, which have been found to upregulate the expression of STAT3 [128]. STAT3 was demonstrated to inhibit ferroptosis by directly promoting the transcription of ferroptosis-negative regulators, such as GPX4, SLC7A11, and FTH1 [129]. On the other hand, EBV-miR-BART5-3p not only suppresses p53 expression but also facilitates the degradation of p53 proteins, which consequently downregulates p21 expression [130]. The p53-p21 axis is a common regulator of ferroptosis [60,61]; thus, inhibition of the axis by EBV-miR-BART5-3p may affect the sensitivity of cells to ferroptosis. Notably, the iron chelator deferoxamine (DFO) can effectively induce EBV reactivation by inducing the binding of p53 and HIF-1α to the EBV-BZLF1 promoter [131]. Quercetin induces ferroptosis in a series of cancers, which is more effective in EBV-positive gastric cancer cells than in EBV-negative cells (Table 1) [122,123]. These data suggest that the modulation of EBV-encoded RNAs can alter the sensitivity of EBVaGC cells to ferroptosis, suggesting a potential therapeutic strategy. Furthermore, DFO and quercetin may exhibit synergistic effects on EBVaGC cells, which further promotes ferroptosis.

### 5.3. Diffuse Large B-Cell Lymphoma (DLBCL)

DLBCL is a relatively common malignant lymphoma that occurs more frequently in the elderly. Only about 5–10% of DLBCL cases carry EBV in cancer cells, and EBV-associated cases show a worse prognosis [88]. EBV-EBNA3B mutation was observed in about 50% of DLBCL cases, which may be critical for EBV-driven lymphomagenesis [111].

Sensitivity profiling in 177 cancer cell lines revealed that DLBCL is a cancer subtype susceptible to ferroptosis [14]. Similarly, studies with a mouse lymphoma model demonstrated that DLBCL is sensitive to ferroptosis induction [112]. Dimethyl fumarate (DMF) effectively reduces intracellular GSH levels in DLBCL cells due to its electrophilic properties, which results in lipid peroxidation and ferroptosis (Table 1) [113]. Bromodomain and extra-terminal domain (BET) inhibitors were able to induce ferroptosis in the germinal center B-cell–like (GCB) subtype of DLBCL, and they also synergized with DMF or RSL3 to kill DLBCL cells [114]. The compound APR-246 triggers ferroptosis by inducing p53-dependent ferritinophagy in DLBCL cells [115]. Artesunate (ART) has been reported to induce apoptosis, autophagy, and ferroptosis in DLBCL cells and acts synergistically with ferroptosis inducers [126]. Iron oxide nanoparticles (IONs) have also shown significant tumor inhibitory effects, which have been demonstrated to enhance ferroptosis [128]. A ferroptosis-related risk score model constructed by Weng et al. divides DLBCL patients into high- or low-risk groups. Patients in the high-risk group reveal resistance to ibrutinib treatment and have significantly shorter survival [128]. As the number of EBV-positive DLBCL cases is minimal, these studies were performed in EBV-negative models. The connection between EBV and DLBCL is worth further investigation, as it remains largely unexplored.

### 5.4. Natural Killer Cell and T-Cell Lymphomas (NKTCLs)

NKTCL is a subtype of non-Hodgkin’s lymphoma derived from mature T cells or natural killer cells that is highly aggressive and has a poor prognosis. NKTCL occurs in the nose, oropharynx, and nasopharynx and is usually accompanied by EBV infection at an early stage [88]. EBV infection is considered an etiological factor in the development of NKTCL [129]. The current treatment strategy for NKTCL is still based on radiotherapy and chemotherapy, although some progress has been made in immunotherapy with PD-1 blockers.

It is reported that kayadiol, a diterpenoid extracted from Torreya nucifera, is able to trigger ferroptosis in NKTCL cells. Mechanistically, kayadiol downregulates SLC7A11 and GPX4 expression by using p53 as a key mediator, which is critical to reverse chemotherapy resistance in NKTCL cells (Table 1) [130]. This study reaffirms the above association between p53 and ferroptosis, which describes the possibility of targeting ferroptosis as a therapeutic strategy for NKTCL.

### 5.5. Burkitt’s Lymphoma (BL)

Based on its pathogenic prevalence, BL is roughly divided into sporadic BL and endemic BL. Sporadic BL occurs at a relatively low level and is usually EBV-negative. In sub-Saharan Africa and some other malaria-prone regions of the world, BL is usually EBV-positive, and the incidence is much higher, which is known as endemic BL [98]. Malaria and EBV cooperate to promote endemic BL development. Compared with EBV-negative BL, EBV-positive tumors carry a much greater mutational burden, suggesting the powerful influence of EBV on the genome of host cells [132].

BL cells have limited uptake capacity for cystine, which increases the susceptibility to oxidative stress-induced cell death [133]. Further studies have proved that EBV-positive BL cells are more sensitive to several ferroptosis inducers (erastin, BSO, and ML-210) compared to EBV-negative BL cells [134] (Table 1). Limited SLC7A11-mediated cystine import and GPX4 activity are among the factors that contribute to ferroptosis sensitivity. The blockade of glutamate–cysteine ligase (GCLC), the rate-limiting enzyme in GSH biosynthesis, significantly increases lipid ROS and cell death in EBV-positive BLs, which can be rescued by Ferrostatin-1 (Fer-1), an inhibitor of ferroptosis [134]. All these features indicate that EBV-positive BL is susceptible to ferroptosis. The high prevalence of endemic BL is always accompanied by malaria, and since artemisinin is a vital drug for treating malaria, this has led many researchers to turn their strategy for BL treatment to using artemisinin and its derivatives. Studies have also shown that artesunate, a derivative of artemisinin, impairs BL cell viability [15]. Further investigation of the mechanisms involved revealed a link between artesunate and ferroptosis. CHAC1 is a glutamyl cyclotransferase that leads to a decrease in intracellular GSH, while the silencing of CHAC1 significantly enhanced the resistance of BL cells to ferroptosis. Artesunate may induce ferroptosis in BL cells by activating the ATF4-CHOP-CHAC1 cascade, which is a branch of the unfolded protein response (UPR) pathway in the endoplasmic reticulum [15,135]. Thus, some evidence suggests that inducing ferroptosis may be a viable therapeutic strategy for EBV-positive BL.

## 6. Conclusions

Ferroptosis has emerged as a trending subject of interest in oncology. It has not been extensively studied in EBV-associated tumors so far. The aim of this paper was to discuss the molecular mechanisms and potential role of ferroptosis in EBV-related cancers. Iron, lipid peroxidation, and antioxidant metabolism centered on GPX4 play key roles in ferroptosis [23]. During EBV infection, intracellular metabolism is remodeled, which affects the regulation of ferroptosis [17,134]. Notably, EBV encodes a variety of gene products, which can impact multiple intracellular ferroptosis-related signaling pathways. The majority of these effects enhance the survival of EBV by inhibiting ferroptosis. Therefore, the induction of ferroptosis has potential in the treatment of EBV-associated cancers.

However, many questions remain to be addressed, which include elucidating the mechanisms by which EBV infection impacts cancer cell ferroptosis sensitivity. As EBV may have both anti- and pro-ferroptotic functions, it will be important to determine the overall impact of EBV infection on host cell ferroptosis sensitivity and whether this is context-dependent. The sensitivity of cells to ferroptosis during the early and late stages of EBV infection is different, which may be due to the different roles played by various EBV-encoded RNAs and proteins in different cells, inducing varying sensitivities to ferroptosis. Therefore, further studies still need to provide a comprehensive molecular understanding of EBV infection and ferroptosis. Various compounds, including radiotherapy, can induce ferroptosis in EBV-associated cancer, but it is unclear whether this is related to EBV infection. EBV-positive tumors and EBV-negative tumors possess distinct metabolic mechanisms, which also affect their sensitivity to ferroptosis. This is an issue that requires further research. This review may provide a reference for further research on the mechanisms involved in EBV infection and ferroptosis, as well as the treatment of EBV-associated diseases.

## Data Availability

No new data were created or analyzed in this study. Data sharing is not applicable to this article.
